# LncRNA HOTAIR promotes the proliferation and invasion/metastasis of breast cancer cells by targeting the miR-130a-3p/Suv39H1 axis

**DOI:** 10.1016/j.bbrep.2022.101279

**Published:** 2022-05-18

**Authors:** Wenxing He, Dongmei Li, Xiaofang Zhang

**Affiliations:** a Breast Cancer Center,Jiangxi Cancer Hospital of Nanchang University;Jiangxi Key Laboratory of Translational Research for Cancer,No. 519 East Beijing Road, Nanchang, Jiangxi, 330029, China

**Keywords:** Breast cancer, LncRNA HOTAIR, MiR-130a-3p, Suv39H1, Metastasis

## Abstract

Long noncoding RNAs (lncRNAs) are a group of transcripts, more than 200 bp in size and regulate cell proliferation, differentiation and apoptosis. LncRNA HOX Transcript Antisense Intergenic RNA (HOTAIR) promotes tumor progression and increases cancer susceptibility by regulating microRNA expression and function. HOTAIR regulates miR-130a-3p expression in hepatocellular carcinoma cells. Bioinformatics analysis revealed that Suv39H1 contained a putative binding site for miR-130a-3p. We speculate that LncRNA HOTAIR promotes the proliferation and invasion/metastasis of breast cancer (BC) cells by targeting the miR-130a-3p/Suv39H1 axis. High HOTAIR expression facilitated BC cell growth and metastasis. HOTAIR functioned as a ceRNA by sponging miR-130a-3p and subsequently promoted Suv39H1-mediated AKT/mTOR signaling. Suv39H1 restoration abolished the effects of HOTAIR knockdown on BC cell growth and metastasis. HOTAIR facilitated the Suv39H1-mediated AKT/mTOR pathway by acting as a molecular sponge of miR-130a-3p.Our results provide a better understanding of the interactions of HOTAIR and miR-103a-3p/Suv39H1 in BC and a potential prognostic biomarker and therapeutic target for BC.

## Highlights

• HOTAIR expression is frequently up-regulated in BC.
• HOTAIR promotes BC cell proliferation and metastasis.
• HOTAIR acts as a ceRNA by sponging miR-130a-3p.
• Suv39H1 is a direct target of miR-130a-3p.
• HOTAIR plays a concogenic role in BC via Suv39H1-mediated AKT/mTOR pathway.

## 1 Introduction

Breast cancer (BC) is the most common cancer among women and ranks second among the main causes of tumor-related deaths in women [1]The prognosis of patients with BC remains unsatisfactory worldwide despite advances in diagnosis and combined treatments [2].Long noncoding RNAs (lncRNAs), a group of transcripts, more than 200 bp in size, are involved in molecular diagnosis and therapy [3,4]. LncRNA HOX Transcript Antisense Intergenic RNA (HOTAIR), as an oncogenic *trans*-acting lncRNA, is elevated in multiple cancers [5] and plays a regulatory role in the growth, apoptosis, migration and invasion of carcinoma [6,7].However, the underlying molecular mechanism of HOTAIR in BC remains not yet fully elucidated.HOTAIR regulates miR-130a-3p expression in hepatocellular carcinoma cells [8]. Bioinformatics analysis from TargetScan Human Release 7.2 (http://www.targetscan.org/vert_72/) showed that Suv39H1 contained a putative binding site for miR-130a-3p. In this study, we speculate that LncRNA HOTAIR promotes the proliferation and invasion/metastasis of BC cells by targeting the miR-130a-3p/Suv39H1 axis.

## 2 Methods

### 2.1 1 patients and specimens

Twenty pairs of human BC tissue specimens and corresponding para-carcinoma normal tissue specimens were collected from patients admitted to Jiangxi Cancer Hospital of Nanchang University (Nanchang, China) from January 2018 to May 2021. No patient received radiotherapy and chemotherapy before the operation. Written informed consents were obtained from all patients. This research was approved by the Ethical Committee on Scientific Research of Jiangxi Cancer Hospital of Nanchang University.

### 2.2 Cell culture

BC cell lines SKBR3,MCF-7 and MDA-MB-231 and human normal mammary epithelial cell line MCF10A were obtained respectively from the Cell Bank of Type Culture Collection of Shanghai Institute of Biological Sciences, Chinese Academy of Sciences. All in vitro cell lines were maintained in Roswell Park Memorial Institute-1640 medium (Invitrogen) supplemented with 10% fetal bovine serum (Invitrogen) and antibiotics (100 μg/mL streptomycin and 100 U/mL penicillin, Sigma, St-Louis, MO, USA) in a humidified tissue culture chamber at 37 °C.Cells in the logarithmic growth period were used for subsequent experiments.

### 2.3 Transfection

The lentivector-mediated HOTAIR (overexpression/HOTAIR and silencing sequences/si-HOTAIR) and negative control (Vector and NC-si) were designed by Shanghai Gene Pharma Company (Shanghai, China). BC cells were transduced with lentiviruses in the presence of polybrene (Sigma). The sequences of HOTAIR and Suv39H1 cDNA were PCR amplified using a PCR Master Mix reagent kit (Thermo Fisher Scientific, USA) and then cloned into the pcDNA3.1 plasmid (Invitrogen). MiR-130a-3p mimics, miR-130a-3p inhibitors, and corresponding negative controls were purchased from Shanghai GenePharma Co., Ltd. (China). BC cells were seeded into 96-well plates (4 × 10^4^ cells/well) in accordance with the manufacturer's protocol. Cell transfection was performed using Lipofectamine® 2000 (Invitrogen), and the cells were incubated at 37 °C in 5% CO_2_.

### 2.4 RNA extraction and RT-quantitative PCR

Total RNA from BC tissues, noncancerous tissues, and BC cells was extracted using TRIzol® reagent in accordance with the manufacturer's instructions, which were reversely transcribed using TaqMan™ mRNA or microRNA Reverse Transcription Kit (ThermoFisher Scientific). Based on primers generated by Sangon (Shanghai, China),RT-PCR was performed using SYBR Premix Ex *Taq*I. HOTAIR (Forward, 5′-GGTAGAAAAAGCAACCACGA AGC-3′; Reverse, 5′- ACATAAACCTCTGTCTGTGAGTGCC-3′); miR-130a-3p (Forward: 5′-GATGCTCTCAGTGCAATGTTA-3′;Reverse:5′-CTCTGTCTCTCGTCTTGTTGGT AT-3′).GAPDH(FW:5′-AATGGGCAGCCGTTAGGAAA-3′,RV:5′-GCCCAATACGACCAAATCAGAG-3′),U6(FW:5ʹ-CTCGCTTCGGCAGCACA-3ʹ; RV: 5ʹ-AACGCTTCACGAATTT GCGT- 3ʹ. All samples were prepared in triplicates. The 2^-ΔΔCt^ method was used to calculate the relative RNA expression.

### 2.5 Western blot analysis

Tumor tissues or cells were lysed with Sigma-Aldrich RIPA buffer. Total protein was quantified by using a BCA protein assay kit (Abcam). Equal amounts of protein samples were separated by 10% SDS-PAGE gel and then transferred onto polyvinylidene fluoride membranes (Millipore, Billerica, MA, USA). The membranes were subsequently incubated with primary antibodies overnight at 4 °C and then incubated with secondary antibodies for 1 h at room temperature.Then, the membranes were visualized with electrochemiluminescence reagents and imaged. The results were analyzed by Image J version 1.8.0 (National Institutes of Health).

### 2.6 Cell proliferation assay

Cell proliferation was evaluated using Cell Counting Kit-8 (CCK-8) assay in accordance with the manufacturer's instructions. CCK-8 reagent was added to the culture medium, and the transfected BC cells in a 96-well plate were incubated at 37 °C for 3 h. Absorbance at 450 nm was recorded for each experiment and normalized to control by using a spectraMax Gemini-XPS microplate reader (Molecular Devices, USA). The optical density ratio of each well indicated cell vitality. The entire experiment was repeated three times.

### 2.7 Colony formation assay

BC cells in the logarithmic growth phase were seeded in six-well plates at a density of 1200 cells/well and then cultured a humidified incubator at 37 °C. After 2 weeks incubation, the cells were washed and fixed in 4% fixative solution (Solarbio, China) for 15 min. The number of visible cell colonies was counted to evaluate cell proliferation.

### 2.8 Transwell invasion and migration assays

Cell migration and invasion were assessed using *trans*-well filters (8 μm pores; Corning, Corning, NY, USA) coated without or with Matrigel (BD Biosciences, NJ, USA) in accordance with the manufacturer's instructions. After transfection, the BC cells were suspended in serum-free medium in the upper chambers. Medium supplemented with 10% fetal bovine serum was added into the lower chamber. After incubation at 37 °C for 12–24 h, the cells on the upper surface of the filters were removed with a cotton swab. The cells underside of the membrane were fixed and dyed on the lower surface of the membrane with hematoxylin and eosin. The membrane was photographed using a colored digital camera connected to a microscope. For migration assay, similar steps were conducted but with the absence of Matrigel coating.

### 2.9 Tumor xenograft model

After the cells were stably transfected with si-HOTAIR or NC-si, SKBR3 cells were injected subcutaneously into the mammary fat pad of 6-week-old BALB/c nu/nu female mice (1 × 10^6^ cells per injection, n = 5 per group). The mice were used according to the guidelines of the Chinese Medical Research Commission and the experiments were approved by the Ethical Committee on Scientific Research of Jiangxi Cancer Hospital of Nanchang University (No. 20180314). Tumor growth was monitored by caliper measurements. The length and width of the tumors were measured. Tumor volume was calculated as 0.5 × length × width^2^. The mice were sacrificed 32 days after implantation.

### 2.10 Pull-down assay with biotinylated miR-130a-3p

BC cells were transfected with biotinylated wild-type (wt) miR-130a-3p, mutant (mt) miR-130a-3p, or NC (Ribo Biotech). The biotinylated RNA complex was pulled down by incubating the cell lysates with streptavidin-coated magnetic beads at 4 °C on the rotator overnight in accordance with the manufacturer's instructions. Then, the beads were washed three times with ice-cold lysis buffer and once with high-salt buffer. The bound RNAs were purified using TRIzol for qRT-PCR analysis.

### 2.11 Luciferase reporter assays

The 3′-UTR sequences of Suv39H1 and HOTAIR were cloned and inserted into the psiCHECK2 vector encoding dual luciferase. The potential miR-130a-3p binding sequences were mutated using the QuikChange® Site-Directed Mutagenesis Kit (Santa Clara). The wt (mt) 3′-UTR of the Suv39H1 vector or wt (mt) HOTAIR vector and miR-130a-3p or control mimics were co-transfected into SKBR3 cells. Luciferase activity was determined using GeneCopoeia's Luc-Pair™ Duo-Luciferase HS Assay Kit.

### 2.12 RNA immunoprecipitation (RIP)

RNA immunoprecipitation (RIP) assay was performed using the Thermo Fisher RIP kit (Thermo Fisher Scientific, MA, USA) in accordance with the manufacturer's protocol. SKBR3 cells were lysed using the complete RIP lysis buffer, and magnetic beads were pre-incubated with a human anti-AGO2 antibody or with a negative control normal mouse *anti*-IgG. RNA was then purified and detected through qRT-PCR.

### 2.13 Statistical analysis

Data are presented as mean ± SD from three independent experiments and analyzed using GraphPad Prism 6.0 Software. Statistical difference was measured using Student's *t*-test or one-way ANOVA. Overall survival curves were protracted using the Kaplan–Meier method and estimated by the log-rank test. Statistical significance was considered at p < 0.05.

## 3 Results

### 3.1 Levels of lncRNA HOTAIR and Suv39H1 are increased in BC

**Fig. 1:**
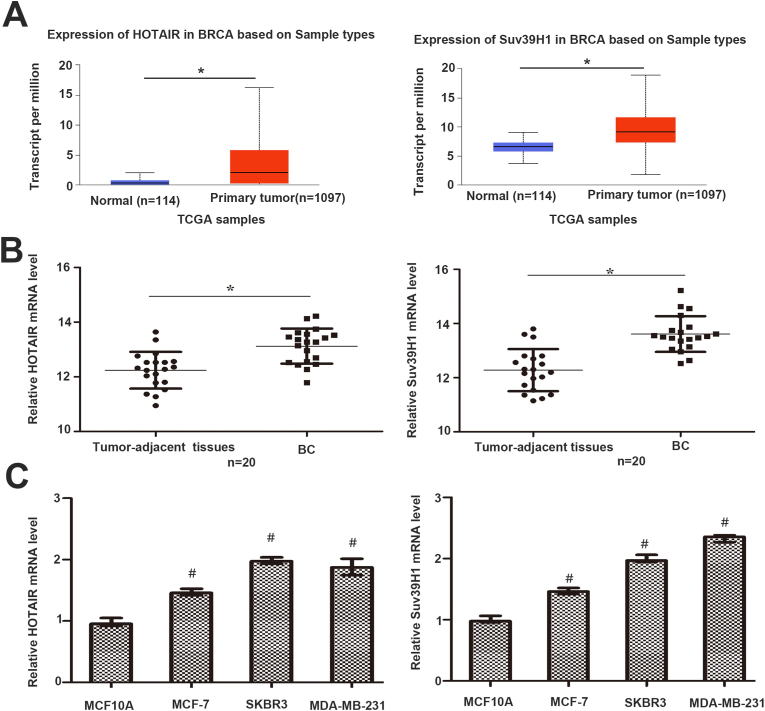
The expressions of HOTAIR and Suv39H1 were highly expressed in BC. (A) TCGA data from UALCAN showed that the levels of HOTAIR and Suv39H1 were increased in BC compared to cancer-adjacent normal breast tissues.(B) The levels of HOTAIR and Suv39H1 in 20 pairs of BC and matched noncancerous tissues were measured by qRT-PCR. HOTAIR and Suv39H1 mRNA in BC were higher than those in tumor-adjacent normal breast tissues. (C) The levels of HOTAIR and Suv39H1 mRNA in BC cell lines (SKBR3 cells,MCF-7 cells and MDA-MB-231 cells) were enhanced compared to normal breast cell line MCF10A, detected by using qRT-PCR. (*p < 0.05 by *Student's t-test,*^*#*^*p < 0.05, vs MCF1*0A by *ANOVA. n=three independent repeats.)*.

The Cancer Genome Atlas (TCGA,http://ualcan.path.uab.edu/analysis.html) data from UALCAN were employed to investigate differentially expressed LncRNA HOTAIR and Suv39H1 in breast carcinoma. Bioinformatics analysis revealed that the expressions of HOTAIR and Suv39H1 were higher in tumor tissues than those in noncancerous tissues(Fig. 1A). The expressions of HOTAIR and Suv39H1 in BC cell lines and 20 pairs of BC and matched tumor-adjacent normal tissues were measured using qRT-PCR to verify the clinical significance of LncRNA HOTAIR and Suv39H1 in BC. HOTAIR and Suv39H1 markedly increased in BC (Fig. 1B-C). These results indicate that HOTAIR and Suv39H1 are highly expressed in patients with BC.

### 3.2 LncRNA HOTAIR and Suv39H1 expression levels reveal poor prognosis of patients with BC

**Fig. 2:**
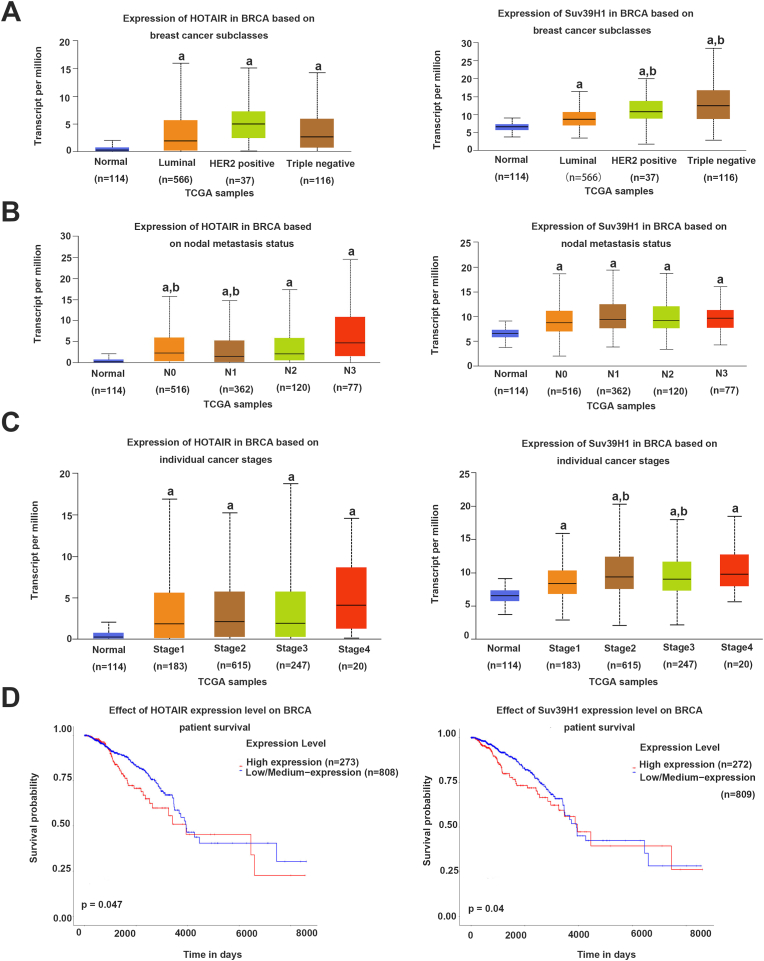
The clinical significance of HOTAIR and Suv39H1 in BC using the TCGA data from UALCAN. (A) The levels of HOTAIR and Suv39H1 in breast tissue with different cancer subclasses and normal breast tissues. ^a^*p* < 0.05 vs Normal breast tissues by ANOVA,^b^*p* < 0.05 vs. Luminal by ANOVA. (B) The expressions of HOTAIR and Suv39H1 in BC based on nodal metastasis status. ^a^*p* < 0.05 vs Normal breast tissues by ANOVA,^b^*p* < 0.05 vs N3 by ANOVA. (C) The expression of HOTAIR and Suv39H1 in BC based on individual cancer stages. ^a^*p* < 0.05 vs Normal breast tissues by ANOVA,^b^*p* < 0.05 vs Stage1 by ANOVA. (D) The effect of HOTAIR and Suv39H1 expression level on BC patient survival. BC patients with high levels of HOTAIR and Suv39H1 had a significant worse overall survival compared to cases with low/medium level of HOTAIR and Suv39H1. BRCA: breast invasive cancer.

BC has four subtypes, namely, HR+/HER2-(“Luminal A″), HR+/HER2+ (“Luminal B″), HR-/HER2-(“Triple Negative”), and HR-/HER2+ (“HER2-enriched"). TCGA indicated that HOTAIR and Suv39H1 were highly expressed in the patients with different cancer subclasses/different nodal metastasis status/individual cancer stages (p < 0.05, vs Normal breast tissues by ANOVA, Fig. 2A–C). HOTAIR was elevated in the patients with N3 (P < 0.05 vs N0-1 by ANOVA). Suv39H1 also increased in the patients with Triple Negative and HER2-enriched (P < 0.05 vs. Luminal by ANOVA)/Stage 2–3 (p < 0.05, vs. Stage 1 by ANOVA, Fig. 2A and C). Survival analysis revealed that the BC patients with high HOTAIR/Suv39H1 expression had a significantly worse overall survival than those patients with low/medium HOTAIR/Suv39H1 expression (Fig. 2D). Collectively, high HOTAIR/Suv39H1 expression is associated with the poor prognosis of patients with BC.

### 3.3 HOTAIR/Suv39H1 facilitates BC cell proliferation, invasion, and metastasis

**Fig. 3:**
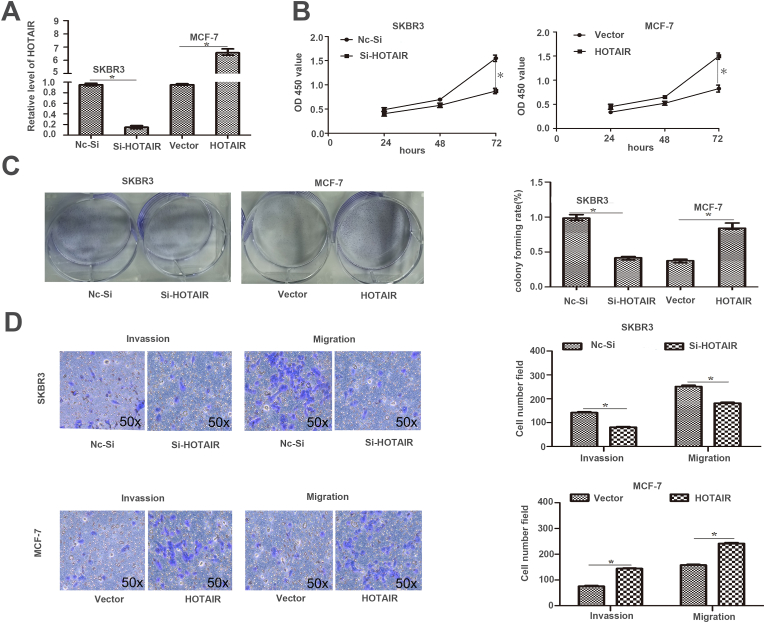
HOTAIR facilitates cell proliferation, migration and invasion of BC cells. (A) HOTAIR was knocked down and overexpressed in SKBR3 and MCF-7 cells, respectively. (B) CCK-8 assay showed that HOTAIR silencing repressed whereas forced expression of HOTAIR elevated BC cell proliferation. (C) The number of BC cell colonies decreased after HOTAIR silencing and increased by HOTAIR overexpression. (D) HOTAIR silencing slowed the process of invasion and migration in SKBR3 cells, while HOTAIR overexpression promoted MCF-7 cell mobility. (*p < 0.05 by *Student's t-test,*^*#*^*p < 0.*05 by *ANOVA . n=three independent repeats.)*.

The lentivector-mediated HOTAIR was used in BC cells to determine the role of HOTAIR in BC. The levels of HOTAIR were downregulated in SKBR3 cells and upregulated in MCF-7 cells, respectively (p < 0.05, Fig. 3A). CCK-8 and colony formation assays showed that HOTAIR depletion significantly inhibited the proliferation of SKBR3 cells, whereas HOTAIR overexpression promoted the proliferation of MCF-7 cells (p < 0.05, Fig. 3B–C). Transwell invasion and migration assays indicated that silencing HOTAIR noticeably suppressed whereas HOTAIR overexpression increased the migration and invasion of BC cells (p < 0.05, Fig. 3D). The results indicate that HOTAIR facilitates the growth and metastasis of BC cells in vitro.

### 3.4 Silencing HOTAIR inhibits cancer proliferation/metastasis and decreases Suv39H1 expression in vivo

**Fig. 4:**
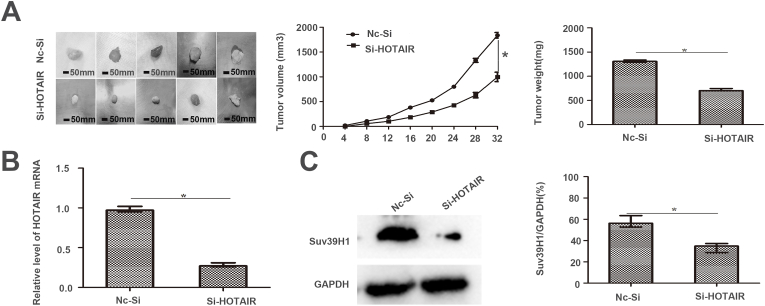
The effect of si-HOTAIR or NC-si on the growth of SKBR3 in xenograft tumors. (A)Both tumor volume and tumor weigh indicated that HOTAIR silencing resulted in reduction of tumor growth in mice. The effect of si-HOTAIR or NC-si on the growth of SKBR3 in xenograft tumors. Dynamic volume of xenograft tumors at different times after injection. Weight of xenograft tumors at the 32nd day after injection.(B)Different expressions of HOTAIR in xenograft tumors derived from SKBR3 cells.(C)The expression levels of Suv39H1 were shown by Western blotting in tumors derived from SKBR3 cells grown in nude mice. HOTAIR silencing inhibited BC growth and metastasis in vivo. (*p < 0.05 by Student's *t*-test, ^#^*p* < 0.05 by ANOVA. n = three independent repeats.)

SKBR3 cells with or without HOTAIR silencing were injected into nude mice subcutaneously to examine the effects of HOTAIR in vivo. Tumor volume and growth were used to observe the tumor growth status. The tumor volume and weight in the HOTAIR silencing group were apparently lower than those in the NC si group (p < 0.05, Fig. 4A). Western blot analysis indicated that Suv39H1 levels significantly decreased in tumor tissues of the HOTAIR silencing group (p < 0.05, Fig. 4B–C). These findings suggest that HOTAIR silencing suppresses the tumorigenesis, growth, and metastasis of BC cells in vivo.

### 3.5 HOTAIR acts as a competing endogenous RNA (ceRNA) by sponging miR-130a-3p

**Fig. 5:**
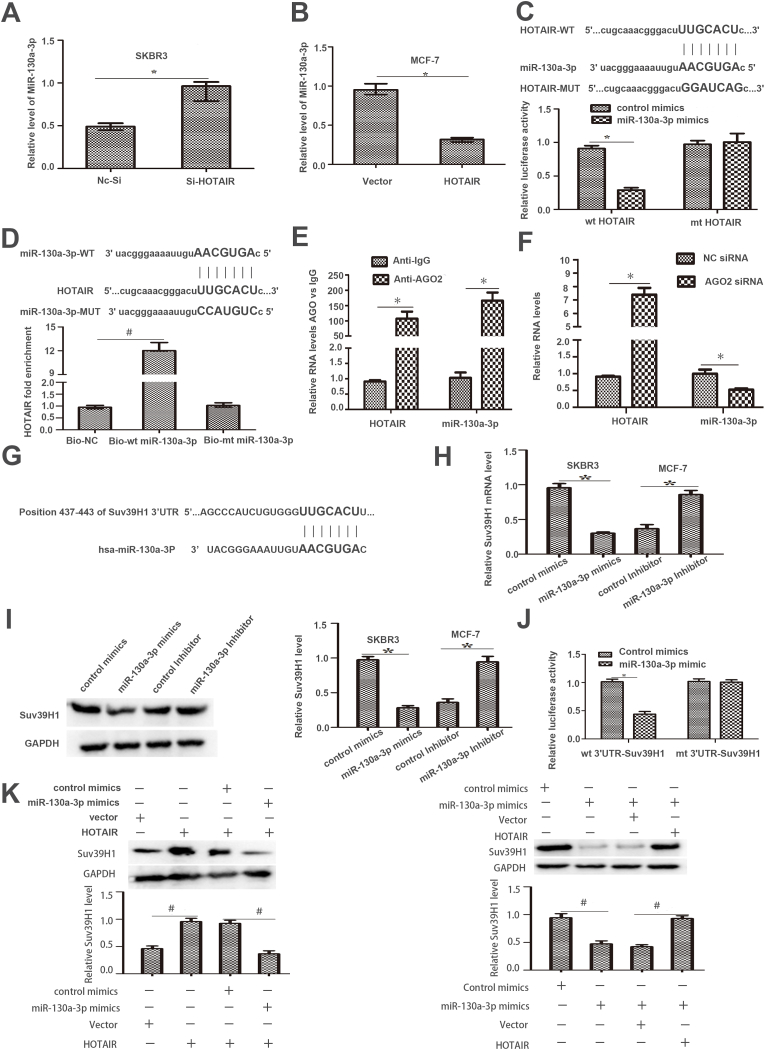
(A–F)HOTAIR acted as miR-130a-3p sponge in BC cells. (A–B) SKBR3 and MCF-7 cells that were transfected with two different si-HOTAIR and HOTAIR vector respectively, were subjected to qRT-PCR for miR-130a-3p expression. HOTAIR down-regulated the expression of miR-130a-3p in BC cells. (C) After co-transfection with miR-130a-3p mimics and vector containing wild type (wt) or mutated (mt) HOTAIR, levels of luciferase activity were measured. MiR-130a-3p mimics transfection inhibited the luciferase activity of the WT but not the MUT HOTAIR reporter in BC cells.(D) HOTAIR was pulled down by biotin labelled wt or mt miR-130a-3p. HOTAIR was highly enriched in the sample pulled down by biotinylated wt miR-130a-3p rather than mt miR-130a-3p. (E). Anti-AGO2 RIP was performed in BC cells, followed by RT-PCR to validate the level of LncRNAHOTAIR or miR-130a-3p associated with AGO2.(F)SKBR3 cells that were transfected with AGO2 siRNA or NC siRNA were subjected to qRT-PCR for HOTAIR and miR-130a-3p expression.(G-K)Suv39H1 is a direct target of miR-130a-3p in BC cells.(G)Binding site of miR-130a-3p and Suv39H1 3′-UTR according to online bioinformatics analysis.(H–I) BC cells were transfected with miR-130a-3p mimics and miR-130a-3p inhibitors, respectively. Suv39H1 expression were detected via qRT-PCR and immunoblotting. (J) Forced expression of miR-130a-3p decreased the fluorescence intensity of the vector containing wild type (wt) 3′UTR of Suv39H1, but did not affect the fluorescence intensity of vector containing mutated (mt) 3′UTR of Suv39H1·(K)Increased expression of HOTAIR enhanced the level of Suv39H1, which was reduced by miR-130a-3p overexpression in BC cells. MiR-130a-3p restoration decreased the expression of Suv39H1, and this effect was reversed by increased HOTAIR in BC cells. (*p < 0.05 by Student's *t*-test, #*p* < 0.05 by ANOVA. n = three independent repeats.).

LncRNAs usually function as ceRNAs and serve as miRNA sponges to inhibit miRNAs [9]. HOTAIR silencing significantly upregulated miR-130a-3p expression, whereas HOTAIR overexpression decreased miR-130a-3p level in BC cells (p < 0.05, Fig. 5A–B). Moreover, luciferase reporter assay indicated that the forced expression of miR-130a-3p strikingly reduced the fluorescence intensity of the vector containing wt HOTAIR rather than the vector containing mt HOTAIR (p < 0.05, Fig. 5C). Furthermore, HOTAIR was pulled down by biotinylated miR-130a-3p, whereas mutagenesis of the binding sites for HOTAIR in miR-130a-3p abolished this interaction (p < 0.05, Fig. 5D). AGO2, as a core element of the RNA-induced silencing complex, acts as an important regulator of miRNA function [10]. The levels of HOTAIR and miR-130a-3p were highly enriched in BC cells, as suggested by the RIP assay using an AGO2 primary antibody (p < 0.05, Fig. 5E). Moreover, AGO2 silencing enhanced HOTAIR expression and reduced miR-130a-3p level in BC cells (p < 0.05, Fig. 5F). These results suggest that HOTAIR acts as a miR-130a-3p sponge in BC cells.

### 3.6 Suv39H1 is a novel target of miR-130a-3p in BC cells

TargetScan was used to predict the miR-130a-3p binding sites in 3′-UTR of Suv39H1 mRNA, and only one potential complementary sequence was found for miR-130a-3p at nt 437–443 (Fig. 5G). Then, the forced expression of miR-130a-3p decreased while miR-130a-3p silencing elevated Suv39H1 mRNA and protein expression in BC cells (p < 0.05, Fig. 5H and I). Luciferase reporter assay indicated that miR-130a-3p overexpression apparently reduced the fluorescence intensity of the vector containing the wt 3′-UTR of Suv39H1 rather than the mt 3′UTR of Suv39H1 (p < 0.05, Fig. 5J). Increased level of HOTAIR enhanced the level of Suv39H1, which was subsequently reduced by the increased level of miR-130a-3p in SKBR3 cells. Forced upregulation of miR-130a-3p remarkably reduced the level of Suv39H1, whereas this effect was reversed by elevated HOTAIR (p < 0.05, Fig. 5K). These results indicate that HOTAIR induces Suv39H1 expression by sponging miR-130a-3p in BC cells.

### 3.7 Suv39H1-mediated AKT/mTOR signal pathway participates in the oncogenic role of HOTAIR

**Fig. 6:**
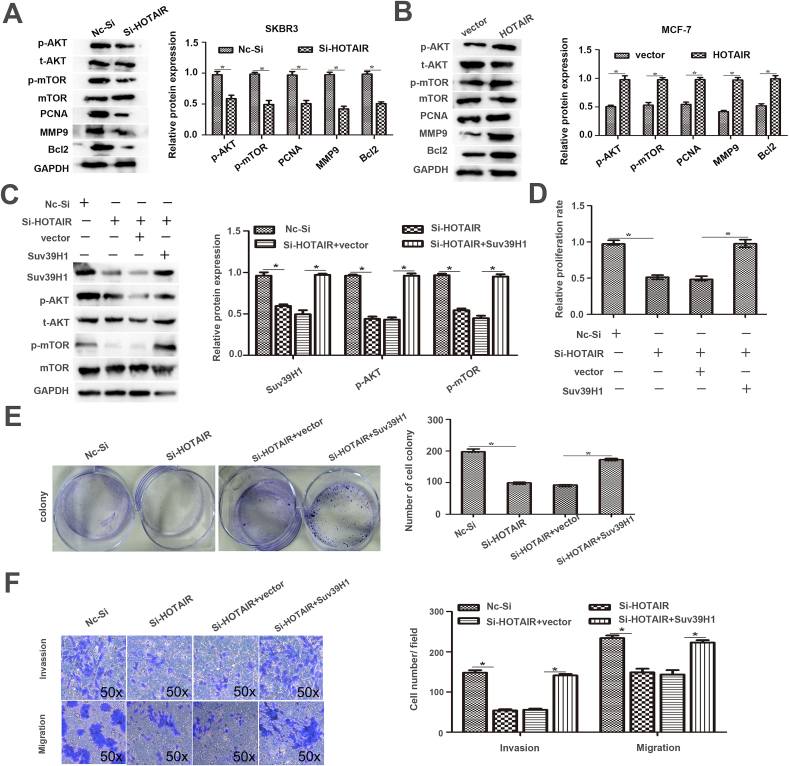
Forced expression of Suv39H1 attenuated the role of HOTAIR silencing in BC cells. (A) HOTAIR silencing down-regulated the expressions of p-AKT, PCNA, MMP9 and Bcl-2 in SKBR3 cells. (B)HOTAIR overexpression enhanced the activation of AKT signaling in MCF-7 cells. (C) SKBR3 cells with HOTAIR silencing that were transfected with Suv39H1 vector or empty vector were subjected to immunoblotting for Suv39H1, p-AKT, AKT, p-mTOR and mTOR expression.(D-F)HOTAIR silencing in SKBR3 cells significantly suppressed cell proliferation, migration and invasion. But Suv39H1 restoration promoted the proliferation, invasion, and migration of SKBR3 cells with HOTAIR knockdown. CCK8(D) and colony formation (E)assays were used to evaluate cell proliferation. Transwell invasion and migration assays(F) were performed to measure cell invasion and migration. (*p < 0.05 by Student's *t*-test, ^#^*p* < 0.05 by ANOVA. n = three independent repeats.)

HOTAIR silencing downregulated the expression levels of p-AKT, p-mTOR, proliferating cell nuclear antigen (PCNA), MMP9, and Bcl-2 in SKBR3 cells (p < 0.05, Fig. 6A). HOTAIR overexpression activated the AKT/mTOR signal pathway in MCF-7 cells (p < 0.05, Fig. 6B). Then, SKBR3 cells with HOTAIR silencing were transfected with Suv39H1 expression vector. Suv39H1 restoration activated the AKT/mTOR pathway in SKBR3 cells with HOTAIR silencing (p < 0.05, Fig. 6C). HOTAIR knockdown inhibited proliferation, invasion, and migration of SKBR3 cells. But Suv39H1 restoration reversed growth and metastasis capacities in SKBR3 cells with HOTAIR knockdown (p < 0.05, Fig. 6D–F). Suv39H1 promotes the proliferation of cervical cancer cells via the AKT pathway [11]. These data reveal that HOTAIR promotes the growth and metastasis of BC cells via the Suv39H1-mediated AKT/mTOR pathway.

## 4 Discussion

Elevated expression of HOTAIR/Suv39H1 was confirmed in our BC tissues and cell lines, which were bioinformatically analyzed in patients with BC (vs. Normal breast tissues). HOTAIR level increased in the patients with N3 (vs N0-1). Suv39H1 expression was enhanced in the patients with Triple Negative and HER2-enriched (vs. Luminal)/Stage 2–3 (vs. Stage1). HOTAIR promotes the proliferation, invasion, and metastasis of human cancer cells [12]. LncRNA HOTAIR indicates its progression and predicates metastasis risk in breast carcinoma [13]. HOTAIR is related to the poor overall survival of patients with ovarian cancer [14]. Collectively, these studies indicate that HOTAIR level increases in human cancer and is related to metastasis and poor prognosis.

Suv39H1 promotes tumor growth in human carcinoma, and pharmacological suppression of Suv39H1 is an effective approach to inhibit human carcinoma [15,16]. Suv39H1 may regulate the proliferation and metastasis of epithelial ovarian cancer cells [17]. Suv39H1 reduces cytotoxic T lymphocyte effector gene expression and causes colon cancer immune escape [18]. Suv39H1 knockdown represses cell proliferation; thus, Suv39H1 might be a promising target for cancer therapy [19].

Subcutaneous tumor formation model in nude mice demonstrated that HOTAIR knockdown inhibited the tumor growth and metastasis of BC in vivo. MiR-130a-3p is decreased in esophageal squamous cell carcinoma and suppresses the migration and invasion of esophageal squamous cancer cells in vitro and in vivo [20]. In the present study, HOTAIR inversely regulated the level of miR-130a-3p in BC cells (Fig. 5A and B). Further experiments identified that HOTAIR acted as a ceRNA by sponging miR-130a-3p. MiR-130a-3p downregulated Suv39H1 in BC cells (Fig. 5H-I). MiR-130a-3p decreased in human BC tissues, and miR-130a-3p in BC stem cells attenuated cellular proliferation, migration and invasion [21]. In the present study, Suv39H1 functioned as a direct target of miR-130a-3p, and HOTAIR enhanced Suv39H1 level by sponging miR-130a-3p in BC cells.

Suv39H1 plays a vital role in the proliferation and migration of prostate cancer cells [22]. Suv39H1 regulates the AKT/mTORC1 pathway in intestinal barrier homeostasis [23]. AKT induces the proliferation, invasion, and migration of cutaneous squamous cancer cells [24]. HOTAIR silencing enhanced the level of miR-130a-3p and reduced the expression levels of p-AKT, p-mTOR, PCNA, MMP9, and Bcl-2 in BC cells (Fig. 5, Fig. 6A). HOTAIR overexpression decreased the level of miR-130a-3p, increased the expression of Suv39H1, and activated the AKT/mTOR pathway in BC cells (Fig. 5, Fig. 6B). MiR-130a-3p overexpression strikingly reduced the levels of Suv39H1 in BC cells (Fig. 5K). Suv39H1 restoration activated the AKT/mTOR pathway in SKBR3 cells with HOTAIR silencing (Fig. 6C).

HOTAIR reduced the level of miR-130a-3p, which inversely regulated the Suv39H1/AKT/mTOR pathway in BC cells. HOTAIR promoted the growth and metastasis of BC cells via the miR-130a-3p/Suv39H1 axis. Our results provide a better understanding of the interactions of HOTAIR and miR-103a-3p/Suv39H1 in BC and a potential prognostic biomarker and more effective clinical therapeutic target for BC.

## Declaration of competing interest

The authors declare no competing financial interests.

## Declaration of competing interest

The authors declare no conflict of interest.

## Data Availability

No data was used for the research described in the article.

## References

[1] Ullah Fahad, Mohammad (2019). Breast cancer: current perspectives on the disease status. Adv. Exp. Med. Biol..

[2] Liang Y., Song X., Li Y., Chen B., Zhao W., Wang L., Zhang H., Liu Y., Han D., Zhang N., Ma T., Wang Y., Ye F., Luo D., Li X., Yang Q. (2020 May 8). LncRNA BCRT1 promotes breast cancer progression by targeting miR-1303/PTBP3 axis. Mol. Cancer.

[3] Rajagopal T., Talluri S., Akshaya R.L., Dunna N.R. (2020 Apr). HOTAIR LncRNA: a novel oncogenic propellant in human cancer. Clin. Chim. Acta.

[4] Finotti A., Fabbri E., Lampronti I., Gasparello J., Borgatti M., Gambari R. (2019). MicroRNAs and long non-coding RNAs in genetic diseases. Mol. Diagn. Ther..

[5] Zhao Wenyan (2018). LncRNA HOTAIR influences cell growth, migration, invasion, and apoptosis via the miR-20a-5p/HMGA2 axis in breast cancer. Cancer Med..

[6] Xia F., Xia W., Yu X. (2020). LncRNA HOTAIR influences the growth, migration, and invasion of papillary thyroid carcinoma via affection on the miR-488-5p/NUP205 Axis. Technol. Cancer Res. Treat..

[7] Ding H., Cui L., Wang C. (2021 Mar). Long noncoding RNA LIFR-AS1 suppresses proliferation, migration and invasion and promotes apoptosis through modulating miR-4262/NF-κB pathway in glioma. Neurol. Res..

[8] Hu M., Fu Q., Jing C., Zhang X., Qin T., Pan Y. (2020 May). LncRNA HOTAIR knockdown inhibits glycolysis by regulating miR-130a-3p/HIF1A in hepatocellular carcinoma under hypoxia. Biomed. Pharmacother..

[9] Lai X.N., Li J., Tang L.B., Chen W.T., Zhang L., Xiong L.X. (2020 Feb 11). MiRNAs and LncRNAs: dual roles in TGF-β signaling-regulated metastasis in lung cancer. Int. J. Mol. Sci..

[10] Ye Z., Jin H., Qian Q. (2015). Argonaute 2: a novel rising star in cancer research. J. Cancer.

[11] Li H., Sun G., Liu C., Wang J., Jing R., Wang J., Zhao X., Xu X., Yang Y. (2017). Suv39H1 is associated with proliferation and poor prognosis in patients with cervical cancer. OncoTargets Ther..

[12] Qu X., Alsager S., Zhuo Y., Shan B. (2019). HOX transcript antisense RNA (HOTAIR) in cancer. Cancer Lett..

[13] Cantile M., Di Bonito M., Cerrone M., Collina F., De Laurentiis M., Botti G. (2020 May 9). Long non-coding RNA HOTAIR in breast cancer therapy. Cancers.

[14] Jiang J., Wang S., Wang Z., Cai J., Han L., Xie L., Han Q., Wang W., Zhang Y., He X., Yang C. (2020 Aug). HOTAIR promotes paclitaxel resistance by regulating CHEK1 in ovarian cancer. Cancer Chemother. Pharmacol..

[15] Lu C., Klement J.D., Yang D., Albers T., Lebedyeva I.O., Waller J.L., Liu K. (2020 Apr 28). Suv39H1 regulates human colon carcinoma apoptosis and cell cycle to promote tumor growth. Cancer Lett..

[16] Takeuchi Y., Tsuge M., Tsushima K., Suehiro Y., Fujino H., Ono A., Yamauchi M., Makokha G.N., Nakahara T., Murakami E., Abe-Chayama H., Kawaoka T., Miki D., Imamura M., Aikata H., Hayes C.N., Tateno C., Chayama K. (2020 Nov 13). Signal activation of hepatitis B virus-related hepatocarcinogenesis by up-regulation of Suv39H1. J. Infect. Dis..

[17] Li J., Shao W., Zhao J. (2021 Mar). MiR-520a-3p inhibits malignant progression of epithelial ovarian cancer by targeting Suv39H1 expression. Hum. Cell.

[18] Lu C., Yang D., Klement J.D., Oh I.K., Savage N.M., Waller J.L., Colby A.H., Grinstaff M.W., Oberlies N.H., Pearce C.J., Xie Z., Kulp S.K., Coss C.C., Phelps M.A., Albers T., Lebedyeva I.O., Liu K. (2019 Mar). Suv39H1 represses the expression of cytotoxic T-lymphocyte effector genes to promote colon tumor immune evasion. Cancer Immunol Res.

[19] Zhao T., Ma X.D., Huang Y.Q. (2013 Jan). [Experimental study of Suv39H1 gene specific siRNA in human leukemia cell line]. Zhonghua Xue Ye Xue Za Zhi.

[20] Tian X., Fei Q., Du M., Zhu H., Ye J., Qian L., Lu Z., Zhang W., Wang Y., Peng F., Chen J., Liu B., Li Q., He X., Yin L. (2019 Mar). miR-130a-3p regulated TGF-β1-induced epithelial-mesenchymal transition depends on SMAD4 in EC-1 cells. Cancer Med..

[21] Kong X., Zhang J., Li J., Shao J., Fang L. (2018 Jun 22). MiR-130a-3p inhibits migration and invasion by regulating RAB5B in human breast cancer stem cell-like cells. Biochem. Biophys. Res. Commun..

[22] Yan W., Guo Y., Xu F., Saxena D., Li X. (2020 Oct 13). Identification of differentially methylated regions associated with a knockout of Suv39H1 in prostate cancer cells. Genes.

[23] Miao Y., Lv Q., Qiao S., Yang L., Tao Y., Yan W., Wang P., Cao N., Dai Y., Wei Z. (2019 Dec 1). Alpinetin improves intestinal barrier homeostasis via regulating AhR/Suv39H1/TSC2/mTORC1/autophagy pathway. Toxicol. Appl. Pharmacol..

[24] Lu D., Sun L., Li Z., Mu Z. (2021 Jan). lncRNA EZR-AS1 knockdown represses proliferation, migration and invasion of cSCC via the PI3K/AKT signaling pathway. Mol. Med. Rep..

