# Supplementary figures and images for: LncRNA HOTAIR promotes the proliferation and invasion/metastasis of breast cancer cells by targeting the miR-130a-3p/Suv39H1 axis
Source: Biochem Biophys Rep. 2022 May 18;30:101279. doi: 10.1016/j.bbrep.2022.101279 (PMC9126846; doi:10.1016/j.bbrep.2022.101279)

1. Figure 4C


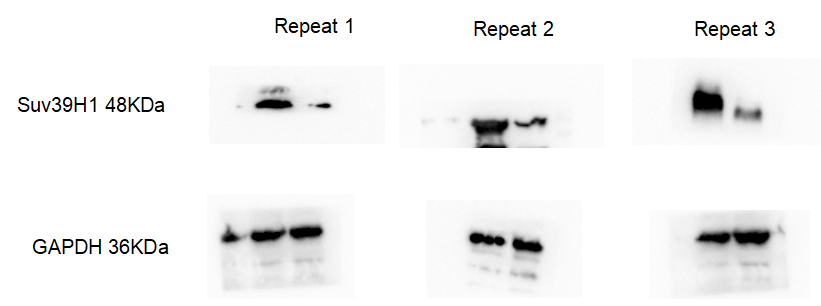


1. Figure 5I


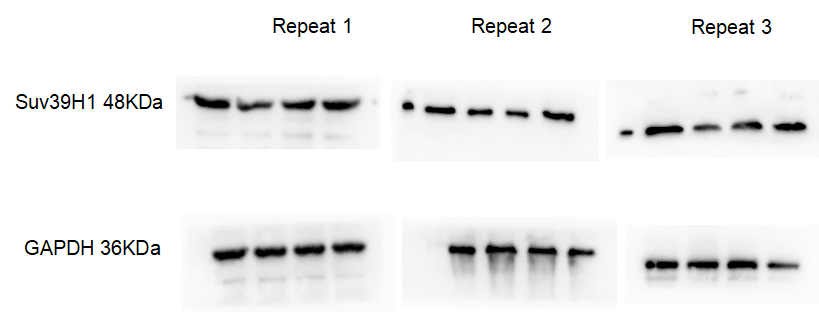


1. Figure 5K


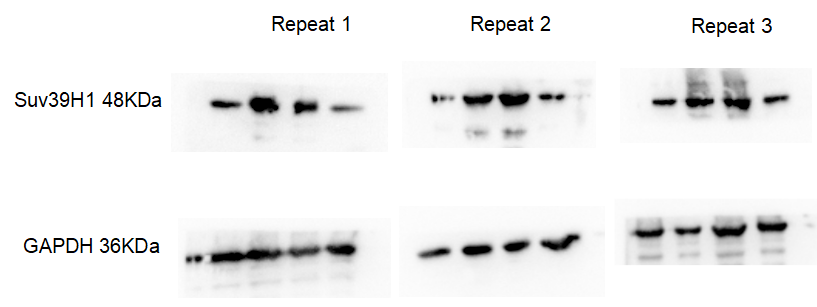


Figure 5K


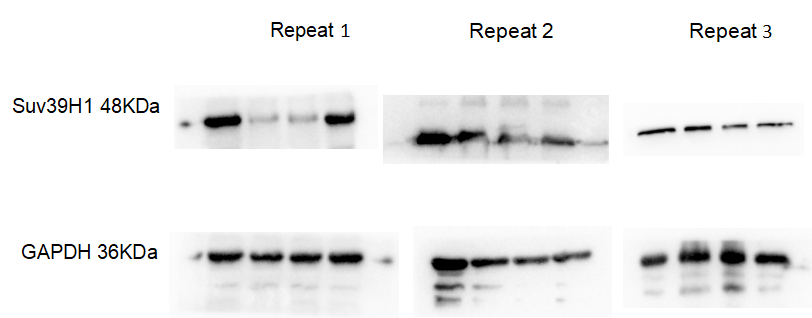


4、Figure 6A


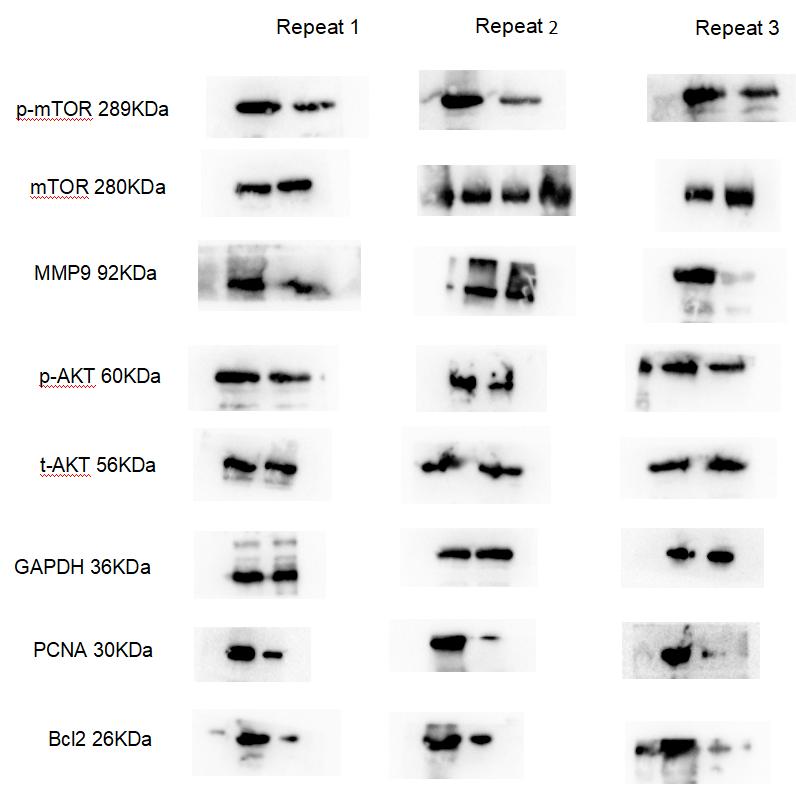


5、Figure 6B


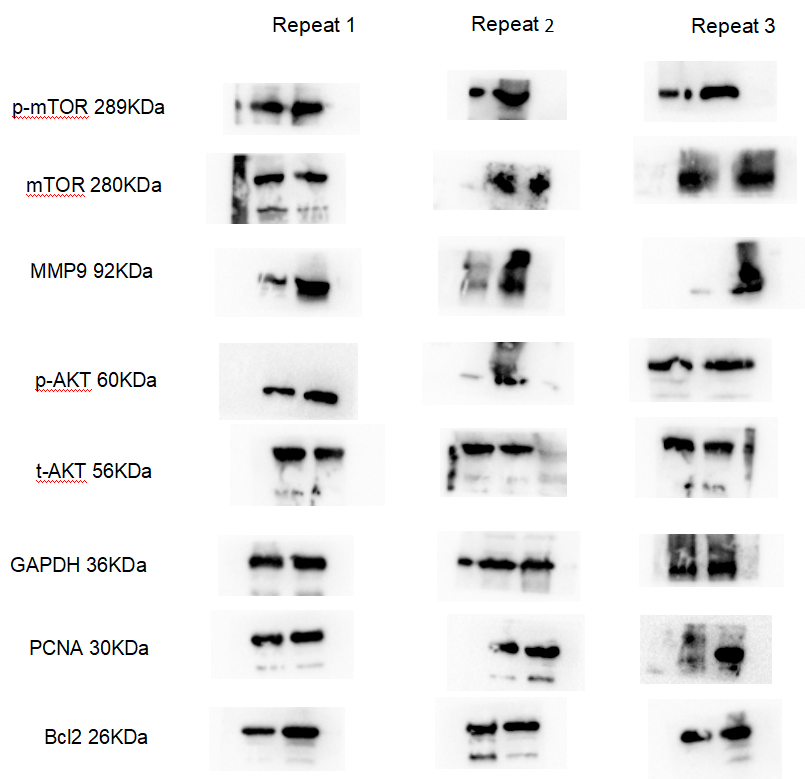


1. Figure 6C
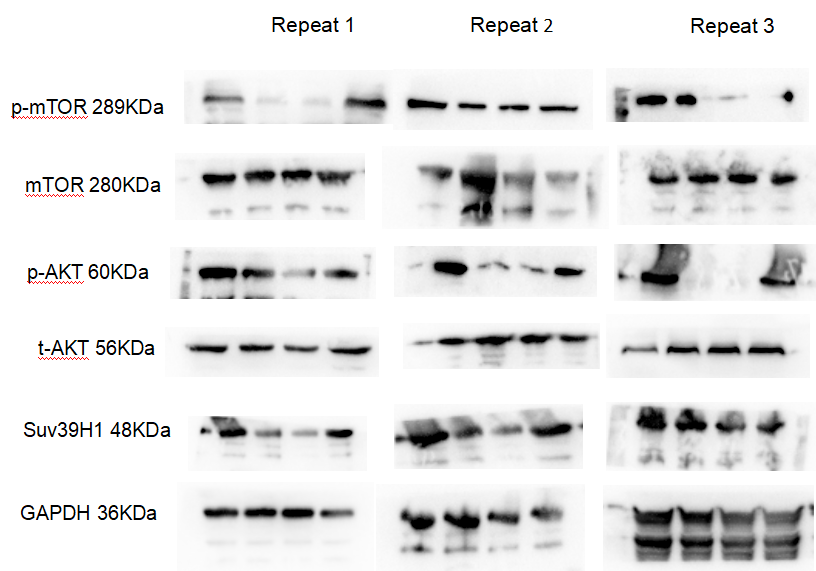

Supplement: Multimedia component 1 [file mmc1.docx]
